# The effects of cerebral oximetry in mechanically ventilated newborns: a protocol for the SafeBoosC-IIIv randomised clinical trial

**DOI:** 10.1186/s13063-023-07699-x

**Published:** 2023-10-28

**Authors:** Maria Linander Vestager, Mathias Lühr Hansen, Marie Isabel Rasmussen, Gitte Holst Hahn, Simon Hyttel-Sørensen, Adelina Pellicer, Anne Marie Heuchan, Cornelia Hagmann, Eugene Dempsey, Gabriel Dimitriou, Gerhard Pichler, Gunnar Naulaers, Hans Fuchs, Jakub Tkaczyk, Jonathan Mintzer, Monica Fumagalli, Saudamini Nesargi, Siv Fredly, Tomasz Szczapa, Christian Gluud, Janus Christian Jakobsen, Gorm Greisen

**Affiliations:** 1 Department of Neonatology, Copenhagen University Hospital ─ Rigshospitalet, Copenhagen, Denmark; 2 Copenhagen Trial Unit, Centre for Clinical Intervention Research, The Capital Region, Copenhagen University Hospital ─ Rigshospitalet, Copenhagen, Denmark; 3 Department of Intensive Care, Copenhagen University Hospital – Rigshospitalet, Copenhagen, Denmark; 4 Department of Neonatology, La Paz University Hospital, Madrid, Spain; 5 Department of Neonatology, Royal Hospital for Children, Glasgow, UK; 6 Department of Neonatology, Children’s University Hospital of Zürich, Zurich, Switzerland; 7 Infant Centre and Department of Paediatrics and Child Health, University College Cork, Cork, Ireland; 8 NICU, Department of Paediatrics, University General Hospital of Patras, Patras, Greece; 9 Department of Pediatrics, Medical University of Graz, Graz, Austria; 10 Department of Neonatology, University Hospital Leuven, Louvain, Belgium; 11 Division of Neonatology and Pediatric Intensive Care Medicine, Center for Pediatrics and Adolescents Medicine, Medical Center ─ University of Freiburg, Freiburg, Germany; 12 Department of Neonatology, University Hospital Motol, Prague, Czech Republic; 13 The Department of Pediatrics, Division of Newborn Medicine, Mountainside Medical Center, Montclair, NJ USA; 14 Fondazione IRCCS Ca’ Granda Ospedale Maggiore Policlinico Milan, Milan, Italy; 15 Department of Clinical Sciences and Community Health, University of Milan, Milan, Italy; 16 St. Johns Medical College Hospital, Bengaluru, India; 17 Department of Neonatology, Oslo University Hospital, Oslo, Norway; 18 II Department of Neonatology, Neonatal Biophysical Monitoring and Cardiopulmonary Therapies Research Unit, Poznan University of Medical Sciences, Poznan, Poland; 19 Department of Regional Health Research, The Faculty of Health Sciences, University of Southern Denmark, Odense, Denmark

**Keywords:** Randomised clinical trial, Near infrared spectroscopy, Protocol, Mechanical ventilation, Brain injury

## Abstract

**Background:**

The SafeBoosC project aims to test the clinical value of non-invasive cerebral oximetry by near-infrared spectroscopy in newborn infants. The purpose is to establish whether cerebral oximetry can be used to save newborn infants’ lives and brains or not. Newborns contribute heavily to total childhood mortality and neonatal brain damage is the cause of a large part of handicaps such as cerebral palsy. The objective of the SafeBoosC-IIIv trial is to evaluate the benefits and harms of cerebral oximetry added to usual care versus usual care in mechanically ventilated newborns.

**Methods/design:**

SafeBoosC-IIIv is an investigator-initiated, multinational, randomised, pragmatic phase-III clinical trial. The inclusion criteria will be newborns with a gestational age more than 28 + 0 weeks, postnatal age less than 28 days, predicted to require mechanical ventilation for at least 24 h, and prior informed consent from the parents or deferred consent or absence of opt-out. The exclusion criteria will be no available cerebral oximeter, suspicion of or confirmed brain injury or disorder, or congenital heart disease likely to require surgery.

A total of 3000 participants will be randomised in 60 neonatal intensive care units from 16 countries, in a 1:1 allocation ratio to cerebral oximetry versus usual care. Participants in the cerebral oximetry group will undergo cerebral oximetry monitoring during mechanical ventilation in the neonatal intensive care unit for as long as deemed useful by the treating physician or until 28 days of life. The participants in the cerebral oximetry group will be treated according to the SafeBoosC treatment guideline. Participants in the usual care group will not receive cerebral oximetry and will receive usual care. We use two co-primary outcomes: (1) a composite of death from any cause or moderate to severe neurodevelopmental disability at 2 years of corrected age and (2) the non-verbal cognitive score of the Parent Report of Children’s Abilities-Revised (PARCA-R) at 2 years of corrected age.

**Discussion:**

There is need for a randomised clinical trial to evaluate cerebral oximetry added to usual care versus usual care in mechanically ventilated newborns.

**Trial registration:**

The protocol is registered at www.clinicaltrials.gov (NCT05907317; registered 18 June 2023).

**Supplementary Information:**

The online version contains supplementary material available at 10.1186/s13063-023-07699-x.

## Background and rationale

Newborn infants can suffer from respiratory insufficiency due to multiple underlying conditions [1, 2]. In preterm infants, the primary reasons are lung immaturity and surfactant deficiency [3], while in infants born at term, reasons may be infection, aspiration of liquor or meconium at birth, persistent pulmonary hypertension, birth asphyxia, congenital malformation, or surgery [4]. Those in need of mechanical ventilation are at high risk of adverse outcomes, not only due to the severity of the underlying condition but also due to complications from the mechanical ventilation itself [1, 2]. Such complications include pneumothorax, ventilation-associated pneumonia, and hyperventilation causing vasoconstriction of the cerebral vasculature and possibly brain ischaemia [5]. Even at term birth, the newborn lungs and heart are immature. Newborn infants have relatively little cardio-pulmonary ‘reserve’-capacity and mechanical ventilation of newborn infants is thus often challenging.

Summary data on outcomes is not easily available for this mixed group of infants. In 10 neonatal intensive care units within the SafeBoosC consortium, death before discharge occurred in 41/500 (8.2%) newborns during 2019 (born at more than 28 weeks of gestation and in need of mechanical ventilation during the neonatal period) (SafeBoosC consortium, unpublished data). A meta-analysis including 895 neonates from 23 retrospective and prospective observational studies undergoing surgery for non-cardiac congenital anomalies found a deficit in intelligence quotient of 0.5 standard deviations (approximately 5 to 7 IQ points) below the population average [6] (although this result is confounded by the risks associated with surgery in itself and those of the underlying conditions). Additionally, data from a Danish national cohort showed that 18% of children who underwent mechanical ventilation during the neonatal period needed special educational support in primary school, which is 2.5 times more often than normal (Wiingreen et al., unpublished data) and the risk of cerebral palsy increased fourfold, after adjustment for other risks [7].

Thus, mechanically ventilated newborns are a high-risk population. Given the instability of the newborn’s pulmonary and circulatory physiology, it is possible that the addition of cerebral oxygenation monitoring by non-invasive near-infrared light technology (cerebral oximetry) plus a treatment guideline, as an addition to the complex treatment and extensive monitoring of these newborns, may increase their chance of surviving without neurodevelopmental impairment [8].

### The preceding SafeBoosC trials

The SafeBoosC-IIIv builds on the execution of the SafeBoosC-II [9] and the SafeBoosC-III trials [10]. The clinical context is that the risk of death as well as the risk of severe brain injury and neurodevelopmental impairment is high in extremely preterm infants and that the transition from intrauterine life constitutes specific risks of hypoxia due to the immature lungs, heart, and vasculature.

The SafeBoosC-II trial evaluated if treatment guided by cerebral oximetry could reduce the time when the brain was hypoxic (or hyperoxic) [9]. Cerebral oximetry by near-infrared spectroscopy was continued for the first three days of life in infants born before 28 weeks of gestational age. A total of 166 infants were randomised over 18 months, across eight European newborn intensive care units. The results showed that it was possible to reduce the time when the brain was hypoxic by more than 50%. Furthermore, there were fewer who died or suffered from severe brain injury in the intervention group, but more intervention group infants suffered from damage to the lungs and eyes. The trial was not powered for clinical outcomes, and indeed none of these differences were statistically significant. Therefore, the consortium was enlarged to conduct the SafeBoosC-III trial, a randomised, phase 3 trial at 70 sites in 17 countries [10]. A total of 1601 extremely preterm infants (gestational age < 28 weeks) were randomised within 6 h after birth, to receive treatment guided by cerebral oximetry for the first 72 h after birth versus usual care. The intervention effect of cerebral oximetry was neutral, as the relative risk for the primary outcome (death or severe brain injury at 36 weeks’ postmenstrual age) was 1.03, 95% confidence interval (CI) 0.90 to 1.18. 

The clinical context of the present SafeBoosC-IIIv trial is intensive care in less immature, although newborn infants. Basically, the brain of all patients that require intensive care is at risk of hypoxic-ischaemic injury, cerebral oximetry by near-infrared spectroscopy is a rational approach to timely detection, and intervention and a trial sequential analysis of 23 trials in children and adults has demonstrated a trend towards benefit, and more than 2300 participants are needed to reach a definitive answer [11]. The SafeBoosC-IIIv, added to the SafeBoosC-III trial, has the potential to do that.

The SafeBoosC consortium presently consists of neonatologists worldwide from over 60 neonatal intensive care units, working together to improve neuroprotective care for critically ill newborns. We here share our plans in the hope that other parties, who consider cerebral oximetry a promising add-on to the monitoring of patients during intensive care, may decide to test the benefits and harms in ways so that the results can be combined.

## Methods/design

### Objective

The objective of the SafeBoosC-IIIv randomised clinical trial is to evaluate the benefits and harms of cerebral oximetry added to usual care versus usual care in mechanically ventilated newborns. The hypothesis is that the intervention will decrease a composite outcome of death or moderate to severe neurodevelopmental disability and/or increase the mean PARCA-R non-verbal cognitive score at 2 years of corrected age.

### Trial design

The trial is an investigator-initiated, multinational, randomised, pragmatic phase III clinical trial. Sixty neonatal intensive care units across 16 countries will be randomising 3000 newborns in total. The trial protocol is in agreement with the SPIRIT guidelines (Table 1) (Additional file 1) [12].

**Table 1:** Schedule for enrolment, intervention and assessment, based on the SPIRIT 2013 guidance for protocols of clinical trials. Asterisk symbol (*) indicates the following: if approved by the local ethics committee, deferred informed consent or prior informed assent may be sought. Time to ask parents for deferred consent will be decided individually by clinical staff members

Visit description	Consent and randomisation	Follow-up #1	Follow-up #2
Visit code	V0	V1	V2
Time period	0–6 h after mechanical ventilation has been initiated	28 days of life	2 years of corrected age
Assessing inclusion and exclusion criteria	X		
Informed consent (can be obtained before initiation of mechanical ventilation)*	X		
Allocation to experimental or control group	X		
Serious adverse reactions (SARs)		X	X
Serious adverse events (SAEs)		X	X
Explanatory variables		X	
Secondary outcomes		X	
Exploratory outcomes		X	X
Neurodevelopmental disability			X
All-cause mortality		X	X

### Inclusion criteria

The inclusion criteria are as follows: newborns with gestational age more than or equal to 28 + 0 weeks, postnatal age less than 28 days, predicted to require mechanical ventilation for at least 24 h (the primary analysis will be ‘as randomised’ thus including infants who are ventilated for shorter times), and signed prior informed parental consent or deferred parental informed consent or absence of opt-out.

### Exclusion criteria

The exclusion criteria are as follows: no available cerebral oximeter, suspicion of or confirmed brain injury, or congenital heart disease likely to require surgery.

### Participation in other trials

Participants may participate in other trials if such this does not interfere with the SafeBoosC-IIIv trial, for example by allowing clinical staff access to cerebral oxygenation values in the usual care group or exclude a treatment in the cerebral oximetry group that would be clearly indicated by the SafeBoosC treatment guideline to reduce cerebral hypoxia.

### Participant discontinuation and withdrawal

The participants’ parents are free to withdraw their infant from the intervention at any time and to decline the use of any future data. If the reason for discontinuation is given by the parents, it will be documented. The attending physician may, in case of safety concerns, withdraw a participant at any time. There are no pre-specified criteria for discontinuation of participants from the trial. Discontinuation of participants will not result in new participants as replacements.

### Recruitment

The feasibility of recruitment evaluating cerebral oximetry combined with the SafeBoosC treatment guideline was proven in the SafeBoosC-II trial [13] and recently in the SafeBoosC-III trial [14]. In the SafeBoosC-III trial, an average of 2.4 newborns were randomised per day (safeboosc.eu). Admission records indicate that the number of potential participants in SafeBoosC-IIIv is larger than for the SafeBoosC-III trial, and new countries and neonatal intensive care units are welcome to join.

### Randomisation

Participants will be randomised through central web-based randomisation. Block randomisation will be used with computer generated varying block sizes unknown to the investigators. Randomisation will be stratified by site, gestational age above or below 34 completed weeks, and expected surgery.

### Blinding

Clinical staff and parents cannot be blinded due to the nature of the intervention. Thus, the primary outcome will not be blinded in participants relying on parental reporting. If there is no contact with the parents, or if they do not return the questionnaire, data will be collected from health care records. All secondary outcomes will be assessed and reported at 28 days after birth by reviewing the newborn’s health care records. Investigators reviewing the health care records will, if possible, be blinded to the allocated intervention.

Data managers, the steering committee, statisticians, and writers of the two final abstracts (one assuming ‘A’ to be the experimental group and one assuming ‘B’ to be the experimental group) before unblinding of the groups will be blinded. The independent data safety monitoring committee will be provided statistics per allocation group named A and B but can request unblinding.

### Intervention

Participants randomised to the cerebral oximetry group will receive cerebral oximetry added to usual care, if possible before tracheal intubation or as soon as possible and within 6 h after mechanical ventilation has been initiated. Cerebral oximetry will continue during care in the neonatal intensive care unit, until the cardio-pulmonary function has been stabilised as indicated by the need for respiratory and circulatory support and evaluated by the responsible physician, until 28 days after birth, or until death. Cerebral oximetry will be used to modify clinical care to reduce cerebral hypoxia, according to the SafeBoosC treatment guideline. Being a pragmatic trial and given the costs and inconveniences of cerebral oximetry monitoring by near-infrared spectroscopy, the responsible clinicians will be free to use, or not use, cerebral oximetry during mechanical ventilation.

Participants randomised to the usual care group will receive usual care without access to cerebral oximetry monitoring.

### Treatment

All treatment options listed in the SafeBoosC treatment guideline are proposals for the responsible physicians on how to support the respiratory and cardiovascular system and keep cerebral oxygenation above the threshold as defined for each type of cerebral oximeter/sensor combination [6].

### Devices

If a given oximeter and sensor combination has been approved for clinical use in newborns and has been calibrated in the blood-lipid phantom [15], it may be used in the experimental group of the SafeBoosC-IIIv trial.

### Trial duration

Recruitment is expected to be completed within 36 months and the primary outcomes known for the last patient 2 years after.

### Outcomes

We will use two co-primary outcomes.

First is a dichotomous composite outcome of death from any cause or moderate-or-severe neurodevelopmental disability at 2 years of corrected age. Moderate-or-severe neurodevelopmental disability will be defined as one or more of the following: (1) cerebral palsy with Global Motor Function Classification System level 2 or higher; (2) the Parent Report of Children’s Abilities-Revised (PARCA-R) non-verbal cognitive function score below − 2 standard deviations (SD); (3) hearing loss corrected with aids or worse; or (4) vision impairment defined as moderately reduced vision of one eye, or only being able to perceive light or light reflecting objects, or blind in one eye with good vision in the contralateral eye. This relies on clinical routine data and the parents’ reports relying on their history with the child. The PARCA-R provides a high test–retest reliability and has been used in multiple clinical trials with 2-year follow-up [16].

Second is a continuous outcome comprising the non-verbal cognitive score of the Parent Report of Children’s Abilities-Revised (PARCA-R), a parental questionnaire, at 2 years of corrected age.

We will use two secondary outcomes.

First is days alive without mechanical ventilation within the first 28 days of life.

Second is one or more serious adverse events within the 28 first days of life. Serious adverse events are defined as one or more of the following: death from any cause, any brain injury diagnosed by imaging, seizures treated with antiepileptic medicine, necrotising enterocolitis defined as Bell’s grade 2 or more [17], sepsis defined as confirmed or suspected infection treated with antibiotics for 5 days or more, extracorporeal membrane oxygenation treatment, renal replacement therapy, use of vasopressor/inotropes, nitric oxygen treatment, and on mechanical ventilation at 28 days of life.

Exploratory outcomes will comprise cerebral palsy defined as Global Motor Function Classification System level 2 or above, at 2 years of corrected age; sensory deficit defined as any degree of vision or hearing impairment, at 2 years of corrected age; all-cause mortality at 2 years of corrected age; use of daily medication during the last 2 months, at 2 years of corrected age; the individual serious adverse events; and days alive without mechanical ventilation within the 28 first days of life. To minimise inter-variance regarding the clinical evaluation of cerebral palsy, vision impairment, and hearing impairment, we will, before initiation of the trial, develop a standard operation procedure with diagnostic criteria to be used by the outcome assessors.

### Statistical plan and data analysis

A fully detailed statistical analysis plan will be developed and published before enrolment is initiated. General principles are outlined below.  

The primary analysis of all outcomes will be based on the intention-to-treat population.

Mixed-effects linear regression and mixed-effects logistic regression will be used to analyse the continuous and dichotomous co-primary outcomes, respectively. In the regression models, we will adjust for the three stratification variables: ‘site’ will be included as a random effect, while ‘gestational age below or above 34 weeks of postmenstrual age’ and ‘group allocation’ will be included as fixed effects. Due to the relatively large sample size, we do not expect major unequal baseline characteristics in the two groups, and therefore, we do not a priori plan to include baseline variables as covariates in the statistical analyses. However, if baseline covariates will be unequal between the two groups, we plan to add sensitivity analyses adjusting for unequal baseline covariates to assess whether such baseline differences lead to different results. To correct for multiple testing, the threshold for statistical significance will undergo Bonferroni adjustment, and thus a *p*-value of 0.025 for each of the primary outcomes is chosen. The superiority of the intervention will only be claimed if at least one of the two co-primary outcomes is statistically significant. All other outcome results will be considered hypothesis-generating only.

### Sample size

To test a reduction in death or moderate to severe neurodevelopment disability from 20 to 16% between the usual care group versus the cerebral oximetry group (a relative risk reduction of 20%), a total of 1500 participants in each group and a total of 3000 participants is needed. This calculation on the dichotomized, composite primary outcome is based on an alpha level of 2.5% and a power of 73%.

### Power of the continuous primary outcome

A power analysis for the continuous primary outcome, i.e. a non-verbal cognitive score of PARCA-R, shows that with a sample of 1500 in each group and an expected rate of death of 8%, and a standard deviation of the PARCA score of 18 points, the power will be more than 98% to detect a difference of 3 points (Cohen’s *d* = 0.2 standard deviation), at an alpha of 2.5%.

### Power of the secondary outcomes

For the first secondary outcome, i.e. days alive without mechanical ventilation within the 28 first days of life with a standard deviation of 15 days (unpublished data) and with a minimal clinical relevance difference of 2 days, the power will be 95% at a 5% significance level.

For the other secondary outcome, one or more serious adverse events within the 28 first days of life as described above, assuming a 50% prevalence among mechanical ventilated newborns and a relative risk decrease of 5% in the experimental group, we will be able to detect this difference between the experimental and control group with 78% power at a 5% significance level.

### Training and certification

Clinical staff will be offered web-based training and certification prior to caring for trial participants. As this is a pragmatic trial, a specific certification rate will not be required before a neonatal intensive care unit can participate in the trial. However, the aim is to achieve the highest certification possible, at least 70% certification proportion in all participating neonatal intensive care units within the first 3 months of inclusion.

Since the web-based training and certification program is a trial quality measure to ensure quality of data and patient care, data on certification rates will be collected and published in a paper investigating the development and implementation of the web-based training and certification program for the SafeBoosC-IIIv trial. The principal investigator at each neonatal intensive care unit is responsible for listing relevant clinical staff who are expected to use the web-based training and certification program, as well as providing trial information, supervision, and support before and during trial conduct.

### Safety

An independent data monitoring and safety committee (DMSC) will be established to monitor mortality and neonatal morbidities at 28 days of life, including serious adverse reactions and serious adverse events. In the SafeBoosC-IIIv trial, serious adverse reactions will be defined as any adverse reaction to the experimental intervention that results in death, is life-threatening, requires hospitalisation or prolongation of existing hospitalisation, and results in persistent or significant disability or incapacity [18], including physical mishaps associated with managing the oximeter and sensors and clinical mismanagement based on data from the cerebral oxygenation monitoring.

The charter for the DMSC will be written prior to inclusion of participants and prior to any analysis.

### Data management

The Copenhagen Trial Unit will provide central, web-based data entry in the electronic case report form (eCRF) using OpenClinica®, an open-source data management environment that was also used for the SafeBoosC-II and SafeBoosC-III trials [19]. The parent report system will use RedCap as developed for the SafeBoosC-III 2-year follow-up study. Data will be managed and stored in line with approval by the Knowledge Centre on Data Protection Compliance, the Capital Region of Denmark.

### Monitoring

Central data monitoring will be performed by the trial manager, the coordinating investigator, and the Copenhagen Trial Unit. Monthly central checks will focus on recruitment to the trial, the quality, completeness, and timeliness of data entry in the electronic case report forms (eCRF). The central data monitoring will be conducted as described by Harboe Olsen et al. [20].

Local Good Clinical Practice monitoring will be done according to the International Conference on Harmonization’s Good Clinical Practice guideline [18]. The following will be monitored locally: all participants for the existence of a clinical file, existence of documented informed consent, and entry of trial participation in clinical files. Source checks will be done for group allocation and survival or death at 28 days of life. The maintenance of delegation and screening logs will be checked.

A screening log will be implemented. All mechanically ventilated infants who are not included will be classified according to cause, date, and counted. The data will be published in the final publication.

### Ethical considerations

Due to the pathophysiology of newborns, the question about cerebral oximetry in mechanical ventilated newborns can only be answered by a trial in this vulnerable population. Currently, there is variation in clinical practice and genuine uncertainty over whether cerebral oximetry is beneficial. There are risks in terms of skin injury and costs in terms of staff time and money for equipment.

The protocol has been approved by the Danish Ethics Committee (H-21071684: July 2022). All neonatal intensive care units must have their eligibility confirmed and the protocol should be approved by the relevant ethics committee before randomisation can begin. All the interventions that are suggested in the SafeBoosC treatment guideline are commonly used in this patient group, and cerebral oximetry is already in routine use in many neonatal intensive care units around the world. Therefore, the methods used to obtain informed consent by parents may be decided by the nationally or locally investigator as prior, opt-out, deferred, or combinations of these methods if it is approved by the ethics committee.

### Publication plan and data sharing

The trial protocol is registered at ClinicalTrials.gov (NCT05907317). Attempts will be made to publish all results, positive, neutral, and negative, in peer-reviewed international journals. Summary data of all outcomes will be uploaded after statistical analyses are completed and publication achieved, if acceptable to the journal to which the manuscript is submitted. If data are not published, we will also upload summary data of entry data, stratification variables, randomisation, and outcomes.

## Discussion

### A window of opportunity

Cerebral oximetry monitoring by near-infrared spectroscopy was brought into clinical routine during cardiac surgery 20 years ago, and despite the lack of large-scale randomised clinical trials proving a clinical benefit, the uptake has also been growing within non-cardiac surgery and neonatal, paediatric, and adult intensive care [11, 21]. New technology has become available, e.g. continuous Doppler ultrasound [22], which is a more direct measure of the clinically ‘missing’ information, i.e. brain blood flow; cerebral oximetry, however, monitored by spatially resolved near-infrared spectroscopy has been thoroughly tested to provide data over many days.

More than twenty randomised clinical trials have evaluated the benefits and harms of cerebral oximetry monitoring, but systematic reviews with meta-analysis in different clinical fields have concluded that the evidence is still insufficient, primarily due to lack of statistical power, poor methodological quality, and a high risk of bias in the published trials [13, 23, 24]. Recently, in a systematic review of the clinical benefits in patients of all ages and in all clinical settings—cardiac and non-cardiac surgery and’medical’ intensive care—with a total of more than 2000 patients, the trend was towards benefit as judged by several outcomes, but the conclusion was that the evidence is still very uncertain [11]. Importantly, heterogeneity among trials was negligible, supporting the approach of merging evidence from all areas of clinical medicine and the concept of ‘hypoxia–ischaemia’ as a threat to all brains.

Since then, the predecessor to SafeBoosC-IIIv—the recently conducted SafeBoosC-III trial [10]—showed no benefit nor risk of serious adverse events in 1600 extremely premature infants.

Cerebral oximetry is an easy add-on to monitoring during intensive care and could potentially help critically ill newborn infants. Although harm seems unlikely, cerebral oximetry does have costs, will cause disturbance for patients, take staff’s time, and will be exposed to the marketing of the device industry. So, adding the SafeBoosC-IIIv trial to the SafeBoosC-III trial, with 3000 and 1600 patients, respectively, will mean that an answer may be reached that is of relevance beyond neonatology. Therefore, the SafeBoosC-IIIv is a highly relevant trial and why a window of opportunity is open now.

We describe here the protocol for a trial to test the hypothesis that monitoring of cerebral oxygenation in newborn infants, as an added element of intensive care during mechanical ventilation, will decrease the composite outcome of death or moderate to severe neurodevelopmental disability and/or the mean of a cognitive score at 2 years of age. We designed a pragmatic trial to make this ambitious trial manageable. In the following sections, the different challenges and countermeasures are discussed.

### Challenge 1: The technology is available; can a relevant benefit be excluded?

The effect size for the sample size calculation is chosen as a 20% relative reduction of the risk of death or moderate-or-severe neurodevelopmental impairment (i.e. 20% to be reduced to 16%). If this effect is demonstrated in the trial, there is little doubt that it will increase the use of cerebral oximetry rapidly. The clinical community is ready, several devices are commercially available, and although the costs are higher than those of pulse oximetry, they are moderate. In the future, costs may even decrease, sensors be improved, and the oximeter signal better integrated into the monitoring systems.

If no effect is demonstrated, however, a question remains whether clinician’s uncertainty about a null effect (since the confidence interval will most likely include a clinically relevant benefit) will make them less likely to start using cerebral oximetry or to stop it if already in use.

The risks of harm seem likely small. In the extremely preterm infant, skin injury and the sheer disturbance caused by the placement and care for the sensor are an issue. However, for bigger infants, this seems less problematic.

In some situations, cerebral oximetry may give much needed information and allow timely and effective clinical intervention. This has been described during extracorporeal membrane oxygenation (ECMO) [25] and during surgery [26]. This line of argument is similar to that behind the use of safety belts in cars, where a small everyday cost and inconvenience may one day be lifesaving. No other tool used to monitor ‘vital signs’ during intensive care, with the potential to reduce the risk of hypoxic-ischaemic brain injury, has been shown to result in patient-relevant benefits in randomised clinical trials [27].

#### Countermeasure

A measure of neurodevelopment was included as a co-primary outcome. The power to detect a small effect will be high. This is not directly patient-relevant, but a null-finding may have more impact on clinical practice.

### Challenge 2: Inclusion and exclusion criteria and generalisability

The SafeBoosC-IIIv trial targets every newborn requiring mechanical ventilation regardless of diagnosis, with some exceptions: extremely preterm infants, as this was the population in the SafeBoosC-III trial, and infants with severe birth asphyxia or other serious brain problems, since the neurodevelopmental prognosis in this group is already serious and this would ‘dilute’ the primary outcomes, although it is far from impossible that cerebral oximetry could also be of benefit in these groups.

Furthermore, infants with congenital heart disease in need of surgery are excluded, since cerebral oximetry is already in use worldwide in this group and it would be more difficult to find equipoise [28]. Similarly, physicians of the other specialties who are involved in the treatment of these infants may or may not use cerebral oximetry routinely and therefore infants who are operated for non-cardiac conditions will not have to follow the protocol while they are cared for by anaesthesiologists and surgeons. This is mainly for pragmatic reasons. It may be too difficult to achieve consensus among colleagues outside neonatology for no-access to cerebral oximetry in the control group, but the price of this may be a reduced potential for the demonstration of benefit.

But even inside neonatology, some colleagues may fail to be in equipoise for some infants. For instance, high risk for ECMO is not an exclusion criterion since cerebral oximetry is not generally used for ECMO, so it is possible that responsible physicians may omit including such infants or withdraw them if deemed necessary.

#### Countermeasure

Screening logs will be required. This will significantly add to the work of primary investigators but will help to quantify the problems regarding generalisability of the results of the trial.

### Challenge 3: Follow-up and risk of missing data of the primary outcome

Both co-primary outcomes are assessed at 2 years of age, when moderate to severe neurodevelopmental impairment can be reasonably reliably identified. The need to trace the participants until 2 years represents another challenge for this trial. With the high number of participants needed and the lack of a major sponsor, it is necessary to design the trial so that it can be integrated into daily clinical practice and would require minimal efforts from clinical staff and minimal data entry from principal investigators who may have no local funding.

Experiences from the ongoing SafeBoosC-III follow-up study (approved protocol at safeboosc.eu), where follow-up is done at 2 years as well, has shown challenges with keeping contact with the parents as well as collection of data from parents.

An advantage for this new SafeBoosC-IIIv trial is that the involvement of the parents in the follow-up process will be in focus from the inclusion in the trial. So, measures will be in place for keeping in contact with the parents, with personal messages and requests for simple information on the health of the child and updates on the progress of the trial. The plan is to create an IT-system to support ongoing contact with the parents throughout the trial to minimise loss-to-follow-up.

#### Countermeasures

A primary outcome at 90 days could be used, and the 2-year follow-up could be secondary. Given the lack of reliable routinely used measures of brain injury in this heterogenous population, the best candidate for this would be ‘days alive out of hospital within 90 days’.

‘High probability of loss to follow-up’ could be used as an exclusion criterium. This was discussed during the draft of the protocol but will not be done, since it would be difficult to operationalise and would reduce the generalisability.

Follow-up could be run by the SafeBoosC project team (central follow-up) to spare the work of principal investigators at each site, but this will involve transfer of patient identity and contact information from sites in all countries to the trial centre in Denmark, which will require separate parental consent and will in principle increase privacy risks.

Some loss to follow-up is unavoidable, and in a 2-year follow-up of a mixed group of newborn infants, this may be significant. The loss to follow-up is likely to be different among children who do well and those who do not. This, however, will not cause bias unless, what is much less likely, the loss is also different among those who had cerebral oximetry and those who did not. A loss to follow-up will always reduce the statistical power. If the loss amounts to as much as 20%, the power of the dichotomous co-primary outcome will be reduced to 62%, and the power of the continuous co-primary outcome will still be more than 95%. One way to account for potential loss to follow-up and thereby missing data for the primary outcome is to increase the original sample size for instance from 3000 to 3750 participants to account for a 20% loss to follow-up. This would increase the statistical power of the primary outcome. However, it is difficult a priori to estimate loss to follow-up. Also, we fear that this could be a pretext for inaction to ‘allow’ missing data during the execution of the trial among the many partners. Additionally, increasing the sample size does not address the risk of bias, which is the ‘true’ problem when loss to follow-up is substantial. To account for missing data in our statistical analysis, we will use the methods described by Jakobsen et al. [29].

### Challenge 4: Blinding

It is not possible to blind the parents or the clinical staff to the intervention. This introduces risks of bias. Mortality by 28 days of life, which is an important part of the primary co-primary outcome, will be determined by source data verification during Good Clinical Practice (GCP) monitoring visits. It is not expected that participation in the trial will influence the parents’ report of the child’s health and development at 2 years of age since it is unlikely to be seen by parents as an important part of their child’s care, although it does not need to be so in all cases.

#### Countermeasures

To quantify the degree of blinding, parents could be asked if they remember if their child had cerebral oximetry or not. This would bring the study purpose back in focus, prompt the parents’ recollections of experimental intervention, and as such potentially increase the bias. Therefore, this will not be done.

### Challenge 5: Data sharing

Given that the SafeBoosC-IIIv trial is a multinational trial, this is an obstacle.

#### Countermeasures

As discussed above, a distributed system will be used, such that patient identity and contact information to parents will be kept at the individual sites, only. But this means that messages to parents must be sent from the individual hospitals. Clinical data will be entered by principal investigators into an end-to-end encrypted web-based participant-record form, identified by a hospital number and a study number, only. Parents, using a QR code, will connect directly to a reporting system in their own language to provide follow-up data.

Although the data kept at the trial centre is still personal, the risk of breach of privacy is reduced as data is pseudo-anonymised. At publication, data used for the analyses will be shared at Zenodo or as instructed by the publishing journal.

### Challenge 6: Collaboration and trial management

Legal work for multinational trials can be extensive. Moreover, it can be hard to keep up spirits in trials that run over several years.

#### Countermeasures

As the SafeBoosC-IIIv trial is pragmatic, there are no intellectual property rights at play, and there will be no economic relation between the sponsor-institution and the hospitals of the principal investigators. Since all aspects of clinical management are decided by the responsible physician, patient insurance will remain the responsibility of hospitals. Thus, contracts only have to cover publication rights and data management.

Effective collaboration between the trial management group and the local investigators over the entire trial period is essential, not least since the primary outcomes will only be complete at ‘last patient out’. We will build on the positive experiences of the SafeBoosC-consortium [9, 10]. National coordinators have the responsibility of ‘hiring and firing’ principal investigators in their respective countries and have a seat in the trial steering group. The trial steering group will meet bi-monthly and have final authority. Trial preparation milestones and recruitment statistics will be compiled monthly and published in a newsletter as a policy of ‘naming and shaming’. The trial management will be by a full time PhD-student paid through central funding. Data management and statistics will be by an academic trial unit, the Copenhagen Trial Unit.

### Challenge 7: Funding—who owns the problem?

The budget for trial management and the trial unit is 1.5 million euros. We applied for funding several times at large research foundations without success despite mature plans and the track record of the SafeBoosC-consortium. This leads to the question of who owns the problem? There is no need for the device industry to provide evidence of benefit, and clinicians are used to apply diagnostic methods without evidence of benefit from randomised trials. Parents are not in a position to question the need. Probably the (public) health sector owns the problem. It is their money, and the time of their staff may be wasted.

#### Countermeasures

Hopefully, funding from such sources can be raised. If only partial funding is obtained, it may be decided to embark on a two-step path, with a primary outcome at 90 days (days alive out of hospital within 90 days) and an adaptive design. Since this is a continuous measure, the statistical power will be higher. With evidence of benefit at an interim analysis, it may be decided to continue to the full sample size, and hopefully it may then be possible to fund the 2-year follow-up.

### Challenge 8: Publication

The success will depend critically on the number and diligence of the principal investigators at each site. The academic ‘pay-back’ is publication credit. It is not doable to write a manuscript among more than 100 authors and really fulfilling the ICMJE-criteria for authorship.

#### Countermeasures

Authorship will be determined by the steering committee according to the Vancouver rules, or they can choose to byline all contributing authors as ‘the SafeBoosC-IIIv group’. All contributions including membership of executive, steering, and writing groups will be detailed in the manuscript.

## Conclusion

In conclusion, there is need for a randomised clinical trial to evaluate cerebral oximetry added to usual care versus usual care in mechanically ventilated newborns.

## Trial status

The protocol is registered at www.clinicaltrials.gov (Version 1.0. NCT05907317; registered 18 June 2023). The protocol is approved by the Danish Ethics Committee (H-21071684: July 2022). Recruitment is expected to begin 1 February 2024 and completed 1 February 2029. Recruitment status can be accessed at www.safeboosc.eu, and interested departments can get in touch using the contact information in the article.

### Supplementary Information

**Additional file 1.** SPIRIT Checklist for Trials.

## Data Availability

Not applicable.

## Abbreviations

PARCA-R: The Parent Report of Children’s Abilities-Revised
SafeBoosC: Safeguarding the Brain of our smallest Children
SafeBoosC-III: Safeguarding the Brain of our smallest Children-III
SafeBoosC-IIIv: Safeguarding the Brain of our smallest Children-IIIv
ECMO: Extracorporeal membrane oxygenation
eCRF: Electronic case report form
SARs: Serious adverse reactions
SAEs: Serious adverse events
